# Academic Nurse Educators' Experiences and Perceptions of Supporting Adult Nursing Students in Clinical Placements in Nursing Homes: An Interpretative Phenomenological Analysis

**DOI:** 10.1111/opn.70076

**Published:** 2026-04-20

**Authors:** Julie Cooke, Helen Aveyard, Kathleen Greenway, Sue Schutz

**Affiliations:** ^1^ Faculty of Health and Life Sciences Oxford Brookes University Swindon UK; ^2^ School of Nursing and Midwifery University of Worcester Worcester UK; ^3^ Oxford School of Nursing and Midwifery, Faculty of Health and Life Sciences Oxford Brookes University Oxford UK

**Keywords:** clinical placement, interpretative phenomenological analysis, nurse educator, nursing home, nursing student, undergraduate nurse education

## Abstract

**Aim:**

This paper explores nurse educators' perceptions of nursing home placements and their experiences of supporting adult nursing students undertaking placements within them.

**Background:**

The global population is ageing and requires the provision of skilled Registered Nurses to meet their needs. However, concerns exist that students do not view nursing homes favourably compared to hospital placements.

**Design:**

Interpretative Phenomenological Analysis.

**Methods:**

Semi‐structured interviews with eight nurse educators were conducted online between December 2020 and March 2021 and transcribed verbatim. The cross‐group analysis elicited individual and shared experiences.

**Results:**

A curriculum focussed on hospital‐based technical skills can result in placement settings such as nursing homes becoming overlooked within nurse education. Participants suggested that student, faculty and nursing home nurses' negative perceptions of the value of nursing home clinical placements make it more challenging to build trusting relationships, and support everyone involved in nursing homes to recognise the potential of their skills and contribution to nurse education.

**Conclusions:**

There is evidence that negative perceptions of nursing home placements are apparent in the nursing faculty and those who support nursing home placements are unwittingly contributing to these negative perceptions, the Registered Nurses who work in them and the skills practised within them. Student nurses are therefore unprepared and unwilling to work in nursing homes, and nursing home staff lack confidence in supporting nursing students. Implementing Care Home Education Facilitators (CHEF) could be a first step to improving this situation. Nurse educators are challenged to ensure the nursing curriculum actively addresses the value of fundamental care throughout the nurse education programme and supports student nurses undertaking clinical placements in nursing homes.

**Implications for Practice:**

Nursing home RNs require better support in their educational roles to improve student experiences of clinical placements in these settings. While technical skills are important for students to learn, overemphasis on them within nurse education programmes can lead to a deficit in the preparation of nurses to deliver fundamental complex care to older people.

## Summary

**What does this research add to existing knowledge in gerontology?:**

- This IPA UK study offers novel insights crucial for global nurse education policy.
- Nursing homes, and the skills of the Registered Nurses (RNs) working within them, are undervalued by faculty and staff delivering care, indicating a systemic undervaluing that goes beyond simple oversight.
- This is exacerbated by poor communication between universities and nursing homes, which impacts effective placement planning, creating significant logistical and relational challenges.

**What are the implications of this new knowledge for nursing care for and with older adults?:**

- Nurse educators observed that nursing home RNs' perceived undervaluation and insufficient support in their educational roles, which reduced their confidence to support students. This directly compromises student experiences.
- Whilst technical skills are important, this focus may lead to a deficit in preparing nurses for the fundamental and complex care needs of older people, which often require a more holistic approach.
- Overemphasis on technical skills can shortchange nurses' preparation for the holistic, complex and fundamental care older people truly need.

**How could the findings be used to influence practice, education, research, and policy?:**

- Nursing education programmes should undertake a review of their curricula to ensure they prepare students for the realities of caring for the ‘oldest old’ in diverse settings, especially nursing homes.
- Universities should provide professional development for academic nurse educators to help them better understand the unique learning opportunities and challenges within nursing home placements.
- Further research is needed to evaluate the impact of the CHEF role on the confidence and competence of RNs supporting adult nursing placements in nursing homes.

## 1 Introduction

The population aged over 65 is rapidly growing and is projected to triple by 2050 (United Nations 2023). Coupled with an increasing number of individuals aged 90 and over in the United Kingdom (Office for National Statistics 2024), this demographic shift is expected to increase the number of older people with complex health and social care needs by 54%, placing greater demands on expert nursing within the social care sector (Age UK 2019). However, In the United Kingdom this demand is challenged by a significant nursing shortage, with Registered Nurse (RN) vacancies in nursing homes at a record high (Royal College of Nursing (RCN) 2022) and a 33% reduction (*n* = 17,000) in nurses working in adult social care since 2013 (Skills for Care 2023).

Nursing, as a practice‐based profession, mandates learning in diverse clinical settings (Nursing and Midwifery Council (NMC) 2023). In the United Kingdom, the NMC stipulates that nurse education comprises 50% theoretical, university‐based learning and 50% clinical practice in various care settings. The effectiveness of these placements is influenced by both the individual student and the learning environment (Jacobssen et al. 2020; Laugaland, Billett, et al. 2021; Dalsmo et al. 2022). The shift towards community‐based healthcare and a reduction of acute hospital beds as per the NHS long term plan (2019) has increased the need for clinical placements outside of the NHS, including in private and voluntary institutions such as nursing homes (Ward et al. 2021).

Despite the large proportion of older people requiring healthcare and the NMC (2018) requirements for students to experience a diverse range of clinical settings, placements in nursing homes specifically for older people are not mandatory for students. Additionally, there is no statutory requirement for content specific to nursing older people in the adult nursing curriculum (Garbrah et al. 2017). This raises a concern that current educational provision may not adequately prepare student nurses for all areas of nursing, as required by pre‐registration education standards (NMC 2018).

The global literature indicates that nursing students often view nursing homes less favourably than acute hospitals for clinical placements (Carlson and Idvall 2014; Ryan et al. 2018; Naughton et al. 2019), perceiving care for older people in these settings as ‘boring and unchallenging’ (Shen and Dongxia Xiao 2012, 221). McCann et al. (2010) suggest that students' perceptions of caring for older people are heavily influenced by the focus of the adult nursing curriculum. These findings imply that nurse education plays an important role in shaping the views of students towards caring for older people in nursing homes. Nurse educators have a responsibility to challenge negative perceptions to prepare students to meet future healthcare demands.

Such negative perceptions can be further reinforced by a lack of role models within the faculty with experience in nursing home care and a curriculum focused primarily on hospital‐based practice and technical skills (Neville et al. 2014; Duggan et al. 2013). This further highlights the significant role nurse educators’ play in shaping student attitudes towards nursing older people in nursing homes and the responsibility of nurse education in driving student interest in specific clinical environments (Duggan et al. 2013). The World Health Organisation (WHO) (2016) recommended that educators develop courses that provide theoretical and practical experiences that focus exclusively on caring for older people. This was to enable students to develop professional identities in nursing homes as well as acute hospital environments.

Cooke et al. (2021) reviewed the literature relating to students' experiences of their placements in nursing homes and concluded that a lack of positive role models impacts students' ability to understand the requirements of the RN in nursing homes. This was also evident in nurse educators' perceptions (Schrader 2009; Laugaland, Billett, et al. 2021; McCloskey et al. 2022), demonstrating that nurse educators are in a strong position to educate students about the role, influence students' clinical placement experiences but also their future career choices (Naughton et al. 2019).

The integrative literature review identified only three papers relevant to faculty perceptions and experiences of nursing homes as clinical placement settings (Schrader 2009; Laugaland, Billett, et al. 2021; Rayner et al. 2022). Schrader (2009) and Laugaland, Billett, et al. (2021) both highlighted university and nursing home management as influential factors on nurse educators' experiences. Specifically, Schrader (2009) reported that positive attitudes and interest from nursing home management in supporting student nurses improved nurse educators' opinions of the learning environment.

Whilst research exists on the use of nursing homes as clinical placement areas and the need for well‐supported placements (Algoso et al. 2016; Keeping‐Burke et al. 2020), there is limited empirical evidence exploring nurse educators' experiences in supporting students undertaking these placements. This study (UK) addresses this gap, identifies strategies for improved clinical practice experiences in nursing homes and raises the profile of this important clinical care setting by exploring educators' experiences.

## 2 Study Aims

This study explored academic nurse educators' experiences and perceptions of nursing home placements and supporting adult nursing students' clinical placements within them.

## 3 Methods

### 3.1 Design

Interpretative Phenomenological Analysis (IPA) (Smith et al. 2009) was used to explore participants' lived experiences and perceptions of supporting student placements in nursing homes. The original Smith et al. (2009) version of IPA was used for this study as the data analysis had already taken place when the methodology was updated in 2022. IPA is distinct from other qualitative approaches as phenomenological, hermeneutic and idiographic components combine to create interpretive understanding (Smith et al. 2022). Throughout the process, the researcher remained aware of their own experiences, opinions and feelings by keeping a reflexive diary, particularly before commencing the interviews, at the end of each interview, and during the analysis process to which they could refer. Smith and Nizza (2022) suggest this is particularly important when the researcher is an ‘insider to the project topic’ (Smith and Nizza (2022), page 17); it can be particularly challenging to separate their own experiences from that of the participants.

### 3.2 Ethical Considerations

The University Research Ethics Committee (UREC) at Oxford Brookes University granted full ethical approval on 11 May 2020. Registration number 291390. This study received no funding.

### 3.3 Recruitment and Participants

Due to the ideographic nature of IPA, Smith et al. (2009, 2022) suggest that a small, homogenous sample of up to 10 participants is suitable for conducting a doctoral study using IPA. Therefore, a sample of eight participants was recruited based on the following inclusion criteria: current or previous experience preparing and supporting adult nursing students for clinical placements in nursing homes, and current or previous employment as nurse educators working with pre‐registration adult nursing programmes in Higher Education Institutions (HEIs). Recruitment involved social media (Twitter, now ‘X’) to identify interested individuals who then completed a brief SurveyMonkey form (version 2020) to provide contact details, consent and confirm eligibility. Eligible participants received an invitation letter, information sheet and consent form. Snowball sampling was also utilised to broaden reach. Participant demographics are presented in Table 1.

**TABLE 1:** Participant‐specific information.

Participant's assigned pseudonym (listed in order of interview)	Registration AN = Adult nurse LDN = Learning disabilities nurse	Type of course delivery	Number of years supporting nursing home placements
1: Sue	AN	Face to face	20 years
2: Jacob	AN	Face to face	4 years
3: Natalie	AN	Face to face	17 years
4: Val	AN	Distance learning	24 years
5: Sam	LDN	Face to face	26 years
6: Kate	AN	Face to face	4 years
7: Jo	AN	Face to face	28 years
8: Maggie	AN	Distance learning	10 years

### 3.4 Data Collection

Due to the COVID‐19 pandemic, interviews were conducted virtually at mutually convenient times. Before each interview, written and verbal consent was obtained from participants. Interviews, which lasted no longer than 60 min, were guided by a flexible interview schedule (see Table 2), developed following two pilot interviews to test the efficacy of the questions, data from which were not used. At the start and end of each interview, the participants and the interviewer chatted about mutually common ground to develop a relationship.

**TABLE 2:** Interview schedule.

Interview questions
How long have you been supporting nursing home placements as a link lecturer?
A recent literature review has highlighted the need for educators and nursing home practice supervisors/assessors to forge effective relationships to recognise environmental differences. To what extent are you able to build effective relationships with practice assessors and supervisors in nursing homes?
Follow‐up questions if needed: What are the barriers to building effective relationships? How do you feel this helps the students to realise their learning opportunities? How do you feel your practice could be enhanced?
The global population is ageing, bringing with it increased frailty and comorbidities. Some of these people will move into nursing homes. In your opinion, how can we begin to facilitate a change in perception towards nursing homes and older people care among faculty staff and nursing students?
How do you feel the adult nursing programme contributes to students' preparation for nursing home placements?
Follow‐up questions if needed: To what extent do you feel this is adequate? To what extent are learning outcomes evident and students informed of possibilities for learning prior to nursing home placements? What would you change about the current preparation for nursing home placements in particular?
A recent literature review has highlighted that some students feel that the curriculum does not prepare them to look after older people. What are your thoughts about this?
To what extent do you feel that a nursing home is able to meet the learning outcomes of students across all years of the programme?

### 3.5 Data Analysis

Online interviews, recorded via Zoom, were transcribed verbatim and analysed using the Interpretative Phenomenological Analysis (IPA) principles of (1) reading and re‐reading; (2) initial noting; (3) developing experiential statements; (4) searching for connections across emergent themes; (5) moving on to the next case and finally (6) looking for patterns across cases (Smith et al. 2009). The analysis employed a double hermeneutic circle, with the researcher using a reflective diary to record personal experiences and understand participants' sense‐making. Emergent themes were developed for each participant's transcript, resulting in eight individual sets of themes. These were then analysed collectively to identify convergent superordinate themes and subthemes. Due to the hermeneutic and ideographic nature of IPA, data saturation was not considered during the analysis process.

## 4 Results

Two superordinate or major themes and six subthemes (Table 3) emerged from the analysis, capturing academic nurse educators' experiences and perceptions of supporting student nurses during nursing home clinical placements. Participant quotations illustrate the idiographic and hermeneutic nature of the analysis, providing insight into their lived experiences. The analysis acknowledges the collective synthesis of themes whilst also emphasising the importance of individual participant voices (Smith et al. 2009).

**TABLE 3:** Superordinate themes and subthemes.

Superordinate (Main) themes	Subtheme
Link lecturers experienced that their workload and a lack of alignment with university processes have an impact on their ability to build a confident community of practice to support nursing home placements	Link Lecturers Perceived a Lack of Confidence in Nursing Home Staff About the Relevance of Their Own Contribution to Student Placements
	Link lecturers experienced additional workload regarding building relationships with nursing homes
	Link Lecturers Perceived a Mismatch Between Nursing Home Practices and University Placement Processes, Finding That NHS Placements Align More Seamlessly
Representing nursing home practice	Nurse Educators Reported a Lack of Strong Faculty and Placement Role Models Possessing Knowledge, Enthusiasm and Awareness of the Unique Aspects of Nursing Home Practice
	Participants Experienced a Change in Students' Attitudes and Perceptions of Fundamental Nursing Care as They Progressed in Their Education
	Participants Perceived That Nursing Older People in Nursing Homes Is Overlooked by the Nursing Curriculum

### 4.1 Theme 1

#### 4.1.1 Link lecturers experienced that their workload and a lack of alignment with university processes have an impact on their ability to build a confident community of practice to support nursing home placements

The findings highlight the participants' views of their role in facilitating student nurse placements in nursing homes. Participants explained their difficulty supporting nursing homes due to organisational structures. This overarching theme is presented in 3 subordinate themes.

#### 4.1.2 Link Lecturers Perceived a Lack of Confidence in Nursing Home Staff About the Relevance of Their Own Contribution to Student Placements

Seven out of eight participants said that they perceived staff in nursing homes to have little confidence in supporting student nurses compared to staff in their other placement settings. Participants said that they worked hard to help nursing home RNs recognise their valuable contribution to nursing education and that this increased their confidence to support students. This is best demonstrated in Maggie's quotation:

getting them [nursing home staff] to see the skills that they have was quite, I think it was probably quite key actually. And I have seen, it has been nice, I mean the relationships have gone through various changes, that you have seen how people [nursing home staff] have gained more ownership of their nursing skills, of their pride in a job, in their supporting of the students and actually, they weren't unsupportive at the beginning, but it was new, and perhaps a bit of fear there. Whereas now, they [nursing home staff] are pleased to see you, they're pleased to have students, they see the development of the students (Maggie, 476–488)

Other participants also spoke about the confidence that nursing home staff need to have in themselves to recognise the value of the skills they have to offer. For example, Jacob mentioned the value of nursing home skills and said:

It might not be that you're the most acutely skilled, but what you have, is you have skills of people and time management, you have skills of delegation (Jacob, 372–375).

Jacob highlighted how he explains to nursing home staff the fundamental skills that they have developed over time, which have become so natural for them that they fail to recognise them as important skills.

#### 4.1.3 Link Lecturers Experienced an Additional Workload Regarding Building Relationships With Nursing Homes

Although all eight participants conveyed certainty that nursing homes are rich in learning opportunities for student nurses if they are well supported by the university, they also experienced reticence from nursing home staff, who did not always feel the same way about their skills. For example, Maggie said:

some of the nurses didn't see how skilled they were in their nursing home environment (Maggie, 100–102).

This implies a lack of value on a wider stage than student placements and the ability to recognise their worth. Additionally, a need to make a particularly special effort to support nursing home staff to recognise their value and role within the sphere of nurse education:

we've got to change the culture of care homes, and we've got to change the culture of care workers and make them feel that their job is valued (Jo, 95–97).

Jo's extract suggested that although all nursing environments need assistance in recognising these important aspects of nurse education, this is particularly evident in nursing homes. This demonstrates that in her experience, nursing home staff feel undervalued compared to staff in other placement settings and that there is an urgent collective responsibility to change nursing home staff perceptions that they cannot support students in the same way as their acute hospital colleagues. The impact of this creates a further divide between the practice areas that link lecturers must consider ensuring that nursing homes are accepted and included in nursing education.

All participants agreed that supporting nursing homes presented greater challenges compared to other care settings, requiring more time and effort to establish trusting and supportive relationships. Five participants attributed this increased challenge to nursing homes being a less common placement area, resulting in nursing home staff having less experience supporting student placements due to lower student numbers, as illustrated by Kate's comment:

I think it's quite difficult for them, and it's challenging for them because they're not sort of dealing with it every day, with 20 students in the way that they would be in a big trust (Kate, 161–164).

This can lead to isolation in nursing homes, impacting both the link lecturer's ability to build relationships and students' interest in these placements. Jacob supported this perspective:

I think there's a bit of a blind spot and I don't know what percentage of nursing homes make up placements, but I guess it's going to be smaller (Jacob, 251–254).

Jacob suggested that lower student numbers translate to lower perceived value from the university, which further isolates the nursing home, the link lecturer and potentially the students placed there. Sue added:

in terms of supporting the nursing home placement generally, they don't fit into the normal model of placement allocations and discussions. In our trusts and acute hospitals and the community trusts there is a much clearer line of communication. (Sue, 28–32).

Sue's belief that nursing homes are not well‐integrated into university communication systems for placement providers, risks marginalising nursing home placements and staff, potentially leading students to undervalue these placements. All eight participants discussed the effort they made outside of their normal link lecturing practice which included being physically visible in nursing homes. Jacob said:

I've had to spend a lot more time going to the homes compared to relying on email or phones to get the answer (Jacob, 34–37).

Additionally, Kate added:

It's often that moment when they're about to fill something in [student paperwork], or do something and they suddenly think, oh crumbs, I can't remember [how to do it] (Kate, 146–147).

Kate emphasises the need for flexibility and immediate availability to nursing home staff, further demonstrating the importance of relationship‐building as a prerequisite for successful placements. This underscores the risk of isolation for nursing home staff supporting students if the link lecturer doesn't make a dedicated effort.

#### 4.1.4 Link Lecturers Perceived a Mismatch Between Nursing Home Practices and University Placement Processes, Finding That NHS Placements Align More Seamlessly

Six out of eight participants reported that NHS trusts (including NHS community placements) possess established infrastructure that facilitates communication between their placement areas and university placement teams, a feature absent in nursing homes. Sue said:

… our acute hospitals and the community trusts have a much clearer line of communication at the placement organisation level (Sue, 35–37).

Sue felt that communication between the university and nursing homes is not streamlined, which directly impacted her support efforts. Kate added clarity to this experience:

We don't have any representation on our practice facilitator meetings from any of the care homes, and that's partly because they don't have practice facilitators (Kate, 175–178).

Kate pointed out the lack of representation from nursing homes, suggesting their perspectives are unheard of due to less frequent contact whilst placement planning. This risks nursing homes being overlooked by university placement teams. Kate emphasised the difficulty of establishing a primary point of contact in nursing homes due to the absence of a dedicated placement representative.

… so the big acute trusts within our area have got teams of practice facilitators. In the nursing home, they don't have that extra layer, and sometimes I find that communication isn't as good (Kate, 123–127).

Kate used the phrase *extra layer of support* to describe the positive impact of facilitators in NHS trusts on communication between the university and placement settings, directly affecting her experience. Jo described feeling isolated when working with nursing homes:

…you just feel, I'm a lone voice (Jo, 513–514).

The lack of dedicated support staff, like practice facilitators or learning environment leads (LELs) who are common in other placement settings, increases the burden on link lecturers. They must work harder to maintain effective communication between the university, students and the placement area.

A contrasting perspective was offered by Maggie, who discussed her experience supporting a nursing home with a dedicated practice facilitator for education and the positive impact this had on communication.

The education lead involved is very protective of those students (Maggie, 541–542).

This extract supports the idea of adding a facilitator role within nursing homes. Val agreed, explaining that a new education facilitator role has been introduced in Wales to enhance communication and the delivery of educational needs for both staff and students in nursing homes.

… the CHEF role… They are the link for the practice experiences in the nursing homes that will close that perceived divide because actually, it will be a health board person that's the practice facilitator in the nursing homes (Val, 540–543).

Val explained how Health Boards in Wales and Scotland are working to improve communication with universities, potentially alleviating the isolation experienced by link lecturers and nursing homes.

### 4.2 Theme 2

#### 4.2.1 Representing Nursing Home Practice

Participants perceived a lack of representation and interest in nursing home practice within programme faculties, which they felt could lead to inadequate representation of fundamental nursing home work in the adult nursing curriculum. This overarching theme is presented in 3 subordinate themes.

#### 4.2.2 Nurse Educators Reported a Lack of Strong Faculty and Placement Role Models Possessing Knowledge, Enthusiasm and Awareness of the Unique Aspects of Nursing Home Practice

Five participants commented on how the prevalence of role models from major hospitals within nursing faculty influences student perceptions of the value of nursing outside of acute and critical care settings. Sam experienced a lack of enthusiasm and knowledge for nursing homes among faculty:

Care homes, and continuing care as I termed it before, I feel that that's missing, and I don't honestly feel there's the appetite or the knowledge [faculty] to provide that (Sam, 414–417).

Participants suggest that whilst their faculty colleagues are experienced nurses, their limited knowledge and interest in nursing homes directly affect students' perceptions of this clinical environment. Jo added her perspective, emphasising the impact that a lack of faculty knowledge and enthusiasm for diverse learning environments can have on student nurses, stating:

If we don't have good role models for [nursing home practice], what can you expect from students? (Jo, 290–291).

Sam shared her experiences of involving faculty without nursing home experience in supporting these clinical placements. She explained:

(faculty) have been given nursing homes as part of their remit to develop, we've got lecturers who came from the very traditional routes [acute practice], and are now involved in getting more PVIs involved [in placements], and as a result of that, changed their perspective (Sam, 664–670).

Exposure to nursing homes as a clinical placement setting has positively impacted the knowledge, understanding and enthusiasm of Sam's faculty colleagues, even those without prior experience in the environment. She added:

All lecturers should have a link [to a nursing home] (Sam, 701–702).

Increased faculty involvement in supporting nursing home placements is expected to raise awareness, knowledge and enthusiasm, ultimately improving both student and link lecturer experiences in this clinical setting.

#### 4.2.3 Participants Experienced a Change in Students' Attitudes and Perceptions of Fundamental Nursing Care as They Progressed in Their Education

Participants noted a change in students' attitudes and perceptions of fundamental nursing care as they progressed in their education. Kate commented:

… [students feel] that what you experience in a care home isn't as important as it is in the hospital (Kate, 246–247).

Additionally, Sam explained the difficulty this causes for her to promote interest in other placement areas outside of the acute hospital setting.

They [students] want to give injections rather than learning to work with human beings and develop therapeutic relationships (Sam, 373–377).

Sam's frustration highlighted students' difficulty in recognising the value of fundamental nursing care in any setting unless it involves performing technical, task‐based skills. Sue connected this to a focus on achieving competencies:

…it's almost like badge collecting. Maybe there's a sense of the more you can do, the more credible you become (Sue, 402–403).

Sue noted that students frequently associate credibility with demonstrating proficiency in tangible, ‘visible’ technical skills. This can lead students to believe that progress is tied to mastering such skills, causing them to seek out placement environments where these skills can be developed. Consequently, nurse educators may prioritise supporting students in these same settings to maximise their chances of success and graduation. Natalie said:

… I think we have to change the mindset of the staff who teach them (Natalie, 408–409).

Natalie suggested that changing faculty attitudes towards nursing home practice is key to broadening students' interest in diverse clinical settings. This implies that evolving nursing roles and misconceptions about nursing home care, possibly reinforced by faculty, are influencing student perceptions. Kate added:

…I think there's always been that kind of Cinderella attitude towards care homes (Kate, 237–238).

Four participants discussed the media's influence on societal and student nurses' perceptions of nursing home placements. Sam said:

…Casualty on a Saturday night, everybody wants to be Charlie Fairhead, everybody wants to work in the A & E department (Sam, 138–140).

Natalie further clarified her point, specifically regarding the media's portrayal of nursing during the COVID‐19 pandemic.

There are people out there with Covid who are not being cared for in ICU, people in nursing homes who've been cared for very well by staff who've not had the luxuries that the NHS has had. You don't have to be in ICU to be looked after with Covid (Natalie, 514–519).

Five participants felt that students prioritise technical skills because the NMC emphasis on technical skills in the standards for pre‐registration nursing education (NMC 2018). Kate said:

a lot of this stigma [surrounding nursing home placements] actually comes from the NMC (Kate, 1033).

Kate expressed concern about the lack of clarity in nurse education policy regarding nursing home opportunities. This ambiguity may lead students and educators to prioritise hospital‐based nursing and its focus on technical skills, potentially undervaluing nursing home practice as a valuable component of nurse education.

#### 4.2.4 Participants Perceived That Nursing Older People in Nursing Homes Is Overlooked by the Nursing Curriculum

This subtheme's findings highlight participants' belief that nurse education needs better integration of nursing homes. They suggested incorporating nursing home placements into theory modules, increasing faculty awareness and mandating more student placements in these settings to showcase their value and prepare nurses for diverse care environments. Sue explained her experiences of the impact she feels this has had on her specific programme's curriculum:

…maybe what we've [education] probably lost sight of is the focus on nursing homes and the uniqueness of the nursing home and caring for the older person (Sue, 188–191).

Sue expressed that the inclusion of nursing home practice will provide knowledge that is not available from any other clinical setting. Sam added:

I think that the emphasis still is more towards preparing students for the more acute environments (Sam, 341–342).

Jacob added that the experience and knowledge of faculty as seen previously, have a direct effect on the examples given to students in class:

I would say there is definitely a tendency and a risk that we default to ward [acute] discussions (Jacob, 225–227).

Jacob highlighted his and his colleagues' tendency to default to acute hospital ward examples due to their familiarity and confidence in discussing these settings. Kate validated this finding when she said:

It's absolutely all acute, isn't it? Acute skills (Kate, 603–604).

Whilst acknowledging that nursing encompasses more than acute care, Kate's emphasis on ‘absolutely’ highlights her experience of the adult nursing curriculum's theoretical focus on acute care, which directly affects her ability to support nursing home placements. Her questioning manner suggests she's seeking confirmation of this observation, perhaps from fellow nurse educators. The most telling aspect might be what she *doesn't* say: any affirmation of a focus on care outside of acute hospital settings.

Jo observed that nurses often return to a focus on fundamental care principles once they realise that task‐based skills are merely a component of delivering excellent nursing care:

No nursing care skill should be less important than another, but certain skills may have to be prioritised over others at certain times yes (Jo, 891–894).

Jo's explanation could imply that nurse education may be prioritising task‐based technical skills over fundamental care, although she acknowledges that this may be necessary at times due to clinical priorities. Natalie captured this when describing clinical skills laboratory teaching:

…deteriorating patient scenarios are about the same patient. There was an A to E assessment, but there's no point at which anybody speaks to the patient (Natalie, 432–435).

Natalie suggested that fundamental care that recognises the person is overlooked, and the importance of technical skills acquisition is placed at the forefront of teaching.

Other participants suggested improving the focus on nursing home care within the adult nursing curriculum. For example, Sue said:

I think we have a responsibility to market nursing home care and care of older people as quite a complex area of practice. That only the best people would be chosen to go and work there, as opposed to the people who couldn't get a job in cardiac intensive care or the emergency department (Sue, 266–272).

Sue acknowledged that specific skills are needed to work in nursing homes and recognises her responsibility to promote nursing homes as environments where students can develop and learn these skills. Jacob suggested that faculty should talk about nursing home care specifically:

So, you can make that a bit more specific and talk about that breadth of experience on an equal level. There's no reason not to exert more examples that are nursing home specific in classroom discussions (Jacob, 322–327).

Jacob discussed the concept of equality, arguing that the knowledge base and learning opportunities available in nursing homes are equally valuable and should be represented and taught as such. Sue added:

…we could talk about the speciality of caring for older people and that working in a nursing home is a speciality choice (Sue, 256–257).

Natalie explained how her faculty has introduced themed pathways. She said:

we've redesigned our placement pathways from September with themed pathways. As many students as possible, they'll have an older person pathway or a care home pathway (Natalie, 140–143).

Natalie emphasised the importance she places on ensuring that all students can experience nursing older people in the nursing home environment. Kate supported this view:

maybe every student should do one care home placement, it should be a compulsory experience (Kate, 1025–1025).

She goes on to explain why:

Because the care of the old person in an acute medical ward is very different to what it is in a care home (Kate, 1029–1031).

Participants felt nursing education overemphasised acute care, neglecting nursing homes and the care of older people. They recommended integrating nursing home placements into curricula, increasing faculty awareness, promoting caring for older people as a speciality and highlighting the unique skills and learning opportunities available in nursing homes.

## 5 Discussion

This research aimed to explore academic nurse educators' perceptions and experiences of preparing and supporting nursing students for clinical placements in nursing homes. The results revealed two key findings: that nurse educators perceived nursing home nurses to lack confidence in their role supporting student nurses on placement. This led to educators reporting the need to make a particularly special effort to support nursing home staff to recognise their value and how they contribute to nurse education. Additionally, results revealed that there is an acute hospital, technical skills focus within the pre‐registration adult nursing programme that directly impacts both student nurse and educator perceptions of the contribution of a nursing home placement.

Whilst nursing homes offer valuable learning environments for student nurses (Berntsen and Bjørk 2010; Carlson et al. 2014; Carlson and Idvall 2014; Watson et al. 2020; Laugaland, Kaldestad, et al. 2021), participants perceived nursing home RNs as lacking confidence in their educational support roles. This aligns with research by Schrader (2009) and Laugaland, Billett, et al. (2021) and suggests a link between nursing home RN confidence and ability to value their role, understanding of programme requirements and academic support. This study emphasises the need for intentional relationship‐building between nurse educators and nursing home RNs, extending beyond simply creating a learning environment.

Boosting RN confidence and competence to support students in nursing homes improves both student placements and resident care (Rivett et al. 2019). Reflective practice, facilitated by academic educators, has been indicated as more effective than teaching alone in increasing staff confidence (Rivett et al. 2019), and collaborative partnerships between universities and nursing homes can clarify learning outcomes and RN contributions to student placements (Suikkala and Kivel 2016). However, Jacobssen et al. (2020) reported inconsistent preparedness by nursing faculty for nursing home RNs to support students on placement. This study highlights the impact of unrecognised needs of nursing home RNs on their perceived ability to support students. Furthermore, it corroborates findings that university faculty may not fully recognise or resource this need (Schrader 2009; Laugaland, Billett, et al. 2021), potentially creating a gap in educational support (Fitzpatrick et al. 2021).

Organisational challenges, including high staff turnover and understaffing in nursing homes (Laugaland, Billett, et al. 2021; Rayner et al. 2022), along with a scarcity of dedicated education facilitators compared to NHS trusts, impede effective communication and the development of strong relationships. Participants from Scotland and Wales emphasised the positive impact of Care Home Education Facilitators (CHEFs), who support learning within care homes by collaborating with nursing home RNs, students and universities to improve the quality of practice learning experiences (Mathisen et al. 2022). Implementing a similar CHEF role in England, modelled after the system in Scotland (NES 2020a, 2020b), could potentially enhance the confidence of nursing home RNs in supporting student nurses. Further research is warranted to evaluate the impact of the CHEF role on RNs supporting adult nursing placements and to determine its applicability in other contexts, including nursing homes internationally.

Research indicates that student nurses often perceive clinical placements in nursing homes as primarily offering opportunities for fundamental nursing care (Brynildsen et al. 2014; Watson et al. 2020), despite the complex needs of older people (Rayner et al. 2022). Participants in this study echoed these perceptions, suggesting that whilst students at a junior level value the focus on basic care often experienced in nursing homes (Ryan et al. 2018; Rayner et al. 2022), this value diminishes as they progress in their education. This aligns with existing literature documenting students' perception of fundamental nursing care as being relevant only for junior staff (Moquin et al. 2018; O'Connell et al. 2020). This perception may imply a perceived lack of knowledge and skills among Registered Nurses (RNs) in nursing homes and an underestimation of their contribution to nursing. Fricker (2007) posits that this creates an epistemic injustice, where the value of nursing home nurses is diminished by the perception that acute hospital experience and technical skills are prerequisites for being a ‘good’ nurse.

Fundamental nursing skills have previously been identified as the essence of nursing care (Kitson et al. 2010; Pentecost et al. 2020; Berman 2022). Alabaster's (2007) study highlighted the perception that nursing older people takes too much time and is the ‘*wrong kind of hard work*’ (Alabaster 2007, 70). Much later, Pentecost et al.'s (2020) literature review of 16 high quality papers similarly found that nurses described a lack of autonomy in prioritising fundamental care above other competing nursing tasks, which gave nurses the impression that they did not have the time to deliver person‐centred hygiene needs. Fundamental nursing care skills therefore seem to be afforded a lower priority than technical task‐based skills and this has a direct impact on the value of a nursing home placement and the confidence that staff in nursing homes have to support student placements.

This study challenges the prevailing emphasis on acute care in adult nursing education, questioning whether it leads to a deficit in the education and representation of nursing home practice. Universities should encourage faculty to leverage their knowledge and experience to address this epistemological gap (Mueler and Travers 2023), benefiting students, educators and nursing home staff who support clinical placements. A comprehensive list of recommendations is provided in Table 4. Neglecting this issue risks further isolating nursing home practice and potentially leaves nursing students inadequately prepared to deliver both fundamental and complex care to the vulnerable population residing in these settings. This study acknowledges that the results are formed based on the experiences and perceptions of 8 nurse educators in the United Kingdom. However, the results contribute to the global knowledge base to promote discussion on nurse education policy and to ensure the future workforce of Registered Nurses is adequately prepared to meet the needs of the population.

**TABLE 4:** Summary of key recommendations arising from this study.

	Recommendations arising from participants' experiences and perceptions of supporting nursing home placements
Nursing policy	Identify throughout nurse education policy and curricula where we pay justice to nursing older people in nursing homes. Redefine what old age means as a first step to providing clarity of the content of nursing older people within the pre‐registration adult nursing curriculum.
Nurse education leadership	Acknowledge and harness stereotypical beliefs and behaviours in nurse education to address implicit ageism. Increase faculty representation (role models) with nursing home experience and knowledge. Nurse education should recognise and understand nursing homes' differences in practice and organisation to enable creative practice. Implement and enhance fundamental care throughout the adult nursing programme.
Practice	Implementation of the CHEF role in England to bridge the gap between education and nursing home practice and encourage the development of double loop learning.
Research	Further evaluation of the CHEF role is required to inform best practice and build a support network to facilitate nurse homes to participate in nurse education. Future studies should consider the impact of an acute hospital care focus within education and if perceptions about what a nurse is and does are changing. Further research studies exploring nursing home staff's perceptions of their professional esteem are required to identify the requirements of nurse education concerning students' understanding of the role of nursing home RN. Future studies to enable innovative change in this area of nurse education should include an examination of the inventory of fundamental care in the adult nursing curriculum in the UK. Students' and educators' perceptions of the efficacy of fundamental skills should be studied, alongside observational studies to address the quality and content of this education.

## 6 Conclusion

This study explored nurse educators' experiences supporting nursing home placements, revealing an epistemic injustice towards nursing homes as learning environments and the skills of their RNs. It calls for open discussion about nurse education's focus and its capacity to prepare a workforce for clinical settings outside NHS trusts. This study offers novel insights for nurse education policy decisions globally, aiding the implementation and support of placements in minority settings like nursing homes. This research empowers nurse educators to advocate for fundamental nursing practice alongside technical skills in future decisions, ensuring that nurses outside majority placement areas are valued for their contributions to caring for the oldest old in settings like nursing homes.

## Author Contributions

**Julie Cooke:** conceptualization, data curation, formal analysis, investigation, methodology, project administration, validation, visualization, writing – original draft, writing – review and editing. **Sue Schutz:** project administration, visualization, director of studies, validation, writing – review and editing. **Kathleen Greenway:** visualization, supervision, validation, writing – review and editing. **Helen Aveyard:** visualization, supervision, validation, writing – review and editing.

## Funding

The authors have nothing to report.

## Ethics Statement

The University Research Ethics Committee (UREC) at Oxford Brookes University granted full ethical approval on 11 May 2020. Registration number 291390.

## Conflicts of Interest

The authors declare no conflicts of interest.

## Data Availability

The data that support the findings of this study are openly available in Radar at https://doi.org/10.24384/hrrf‐4578.
